# Debonding Detection of Thin-Walled Adhesive Structure by Electromagnetic Acoustic Resonance Technology

**DOI:** 10.3390/ma17205073

**Published:** 2024-10-17

**Authors:** Ne Liu, Shiqiang Shen, Ying Zhu, Ying Gao, Yongdong Pan

**Affiliations:** 1 Key Laboratory of Nondestructive Testing, Ministry of Education, Nanchang Hangkong University, Nanchang 330063, China; nliufws@gmail.com (N.L.); ssq_3276@126.com (S.S.); nuaazy06@126.com (Y.Z.); 2 Department of Mechanical Engineering, University of Hong Kong, 7/F Haking Wong Building, Pokfulam Road, Hong Kong, China; gying0223@163.com; 3 School of Aerospace Engineering and Applied Mechanics, Tongji University, 100 Zhangwu Road, Shanghai 200092, China

**Keywords:** electromagnetic acoustic resonance, thin-walled adhesive structure, debonding defect, nondestructive testing

## Abstract

The detection of debonding defects in thin-walled adhesive structures, such as clad-iron/rubber layers on the leading edges of helicopter blades, presents significant challenges. This paper proposes the application of electromagnetic acoustic resonance technology (EMAR) to identify these defects in thin-walled adhesive structures. Through theoretical and simulation studies, the frequency spectrum of ultrasonic vibrations in thin-walled adhesive structures with various defects was analyzed. These studies verified the feasibility of applying EMAR to identify debonding defects. The identification of debonding defects was further examined, revealing that cling-type debonding defects could be effectively detected using EMAR by exciting shear waves with the minimum defect diameter at 5 mm. Additionally, the method allows for the quantitative analysis of these defects in the test sample. Due to the limited size of the energy exchange region in the transducer, the quantitative error becomes significant when identifying debonding defects smaller than this region. The EMAR identified debonding defects in clad-iron structures of rotor blades with a maximum error of approximately 15%, confirming its effectiveness for inspecting thin-walled adhesive structures.

## 1. Introduction

Thin-walled adhesive structures are widely utilized in critical fields such as aerospace and nuclear industries due to their advantages, including lightweight properties, high specific strength, and excellent damping capabilities. Among these, thin-walled metal and non-metal adhesive structures are particularly prevalent in aerospace components due to their corrosion resistance, thermal insulation, and enhanced fatigue resistance. These structures are commonly found in aircraft skins, liners, honeycombs, as well as clad-iron and composite layers, including blades on the leading edges of helicopter rotor blades [1,2].

Debonding is the primary defect encountered in adhesive structures, typically occurring between composite layers and presenting as an area defect. Ultrasonic methods are widely employed to detect such defects [3,4,5,6]. However, there are disadvantages in using serval methods. For contact methods, dry coupling is difficult to manage, especially when using nonlinear methods [4]. While the water immersion technique is efficient, it does not meet the requirements for in situ or large detection [7]. Among non-contact methods, air coupling offers relatively unsatisfying accuracy, and laser ultrasonics poses a risk of damaging objects [8,9]. Unlike traditional piezoelectric ultrasonic, which primarily uses longitudinal waves for detection, electromagnetic acoustic transducers (EMAT) can more effectively generate and receive shear waves, which are more sensitive to the bonding interface condition. EMAT utilizes transducers to generate and receive ultrasonic waves, offering advantages such as non-contact detection, strong environmental adaptability, and no need for coupling agents. There have already been many successful cases of its application in detecting structures that contain metal materials [10,11,12,13,14,15]. Moreover, some theories have been discussed for helping to understand how to capture the electromagnetic response effectively [16,17].

Electromagnetic acoustic resonance (EMAR) detection technology is a novel method that combines the principles of electromagnetic ultrasonic detection with the effects of ultrasonic resonance. Ultrasonic resonance, also known as ultrasonic standing wave resonance, can be considered as the standing wave resonance of body waves in the thick direction of the plate. EMAR technology has been applied for the measurement of metal material dimensions and fatigue damage monitoring [18]. For instance, Heo et al. [19] used EMAR to detect surface cracks approximately 100 μm deep. Cai et al. [20] successfully measured the thickness of inclined metal plate samples and determined the direction of the slope. Additionally, Liu et al. [11] demonstrated the capability of EMAR in addressing the debonding issue of aluminum plates on the surface of plexiglass.

In adhesive structures, changes in the bonding state at the interface led to alterations in the ultrasonic reflection coefficient, affecting the energy intensity of the ultrasonic echo. Therefore, the amplitude of the echo can serve as an indicator of debonding defects. Research has also demonstrated that the reflection coefficient of an interface can be described as a function of the incident wave frequency. As a result, the reflection effects differ across ultrasound frequencies [6]. This variation is most notable near the resonant frequency, where higher reflection coefficients are observed. Furthermore, reflection behavior depends on factors such as wave modes and the angle of incidence [21]. When tight-contact or inclusion-type debonding defects are present, the responses to the incidence of shear or longitudinal waves differ significantly, with shear waves showing a more pronounced effect [22]. Another method to generate shear waves is using obliquely incident longitudinal waves, which relies on wave mode conversion at the interface. However, the detection of the reflected waves requires highly precise angle measurement equipment, making this approach difficult to implement [23]. While EMAR offers an effective solution to these challenges, by adjusting the design of the EMAT and controlling the excitation modules, EMAR can generate ultrasonic waves of various frequencies and modes within the sample. Furthermore, the echo amplitude obtained by EMAR is significantly higher than that obtained by traditional ultrasonic methods. As a result, EMAR has strong potential for inspecting thin-walled adhesive structures, such as the leading edges of helicopter rotor blades.

This study focused on the identification of debonding defects in the clad-iron/rubber layers of thin-walled adhesive structures on helicopter blade leading edges. EMAR detection technology, which combined ultrasonic resonance and electromagnetic ultrasonics, was applied. First, the theoretical basis for EMAR detection technology was introduced. Then, a simulation model was developed to verify the feasibility of using EMAR for detecting debonding defects. An experimental EMAR system was set up to perform qualitative, locational, and quantitative analyses of debonding defects with different bonding states and sizes. Eventually, the method was further validated on the clad-iron structures at the leading edges of the helicopter blades.

## 2. Theories of EMAR

**Figure 1:**
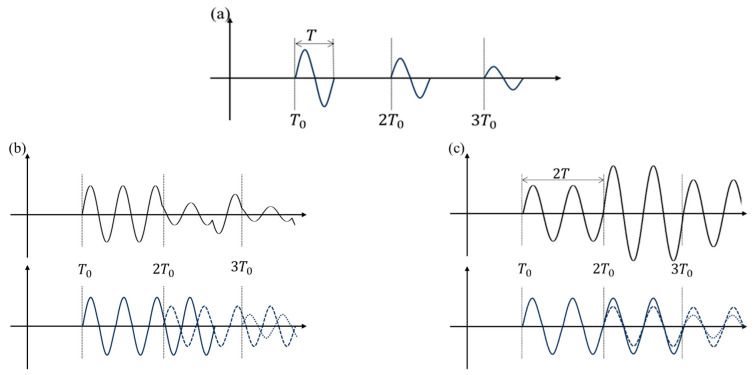
The interference of echoes in the plate. (**a**) Single-period echo. (**b**) Destructive interference with four-periodic echoes. (**c**) Constructive interference with four-periodic echoes.

To explain the theory of EMAR, consider a scenario where a transducer is placed on the surface of a plate with a thickness of *d*, generating a single-cycle ultrasonic wave propagating in the thickness direction. The wavelength and period of the wave are *λ* and *T*, respectively. The pulse wave undergoes multiple reflections between the two free surfaces of the plate, and each time it returns to the incident surface, it is received by the same transducer. The received signal is shown in Figure 1, where the horizontal axis represents time, the vertical axis represents amplitude, and $T_{0}$ denotes the time it takes for the ultrasonic wave to propagate a complete round trip in the thickness direction. Due to wave attenuation within the material, the amplitude gradually decreases as the propagation path increases.

In the case where *λ* ≪ *d*, the received signal is shown in Figure 1a. The echo signal consists of several independent single-period pulses, each of which does not interfere with the others. However, if period T of the excitation signal or the thickness *d* of the plate is adjusted, leading to originally independent signals to overlap, constructive and destructive interference occur in the echo signal. As shown in Figure 1b,c, suppose a pulse signal with four periods is used as the excitation. Before the first echo is fully received, the second echo reaches the incident surface, causing the end of the first echo to overlap with the beginning of the second echo. This pattern of overlap continues between the second and third echoes, between the third and fourth, and so on. In the overlapping regions, phase differences between the echoes, due to differences in propagation distance, lead to destructive and constructive interference. This interference reduces or enhances the signal amplitude in the echo.

It is evident that the degree of interference in the received signal depends on specific parameters of the excitation signal and the plate thickness. When the plate thickness remains constant, the signal interference is determined solely by the excitation signal parameters.

As the ultrasonic waves are repeatedly reflected within the sample, the received time-domain waveform $F\left(t\right)$ can be regarded as the sum of multiple time-domain signals $F_{i}\left(t\right)$. Furthermore, the received multi-echo signal $F\left(t\right)$ can be expressed as a function of the frequency:

(1) $$F(t)=F_{1}(t)+F_{2}(t)+\cdots F_{N}\left(t\right)=\sum_{i=1}^{n}A_{i}(t)\sin(2\pi ft+\varphi_{i})$$

(2) $$\varphi_{i}=\varphi_{1}-(i-1)\times 2\pi ft\times 2d/v,i=1,2,\cdots N.$$

In the formula: $A_{i}\left(t\right)$ represents the envelope of the *i*-th received signal waveform $\;F_{i}\left(t\right)$, *d* is the distance between the upper and lower interfaces, v is the wave velocity, f  is the signal frequency, and $\varphi_{1}$ indicates the initial phase of the excitation signal.

Resonance occurs when the sum of the amplitudes $\bar{A}=\int_{t_{1}}^{t_{2}}A\left(t\right)dt$ reaches its maximum value. At this point, the carrier waves of each time-domain signal in Equation (2) must have the same phase, which is expressed as 2πf×2d/v=2π×n, where *n* is a positive integer. It is noteworthy that in this spatial location, the amplitudes of all waves will be superimposed, yet not all frequencies or phase differences will constitute a complete period. In such instances, the amplitude will cancel out, resulting in attenuation or even a value of 0 (i.e., standing waves). The wave in resonant frequency will get enhanced. The *n*-th resonant frequency of the sample can be obtained as follows:

(3) $$f_{n}=n\frac{v}{2d}$$

The core of EMAR detection technology lies in generating ultrasonic resonance. The technology features an adjustable excitation frequency, allowing the signal frequency to be tuned for specific test objects or applications. By matching the excitation signal frequency with the object’s resonant frequency, a resonance effect is achieved, thereby enhancing the received signal.

In ultrasonic resonance detection of adhesive structures, the resonant waveforms resemble those in double-layer structures. When the bonding is intact, the received signal contains not only the resonant wave modes of the upper layer but also those where resonant waves from the lower layer leak into the upper layer. It also includes resonant wave modes generated by the incident wave and multiple round-trip transmitted waves.

**Figure 2:**
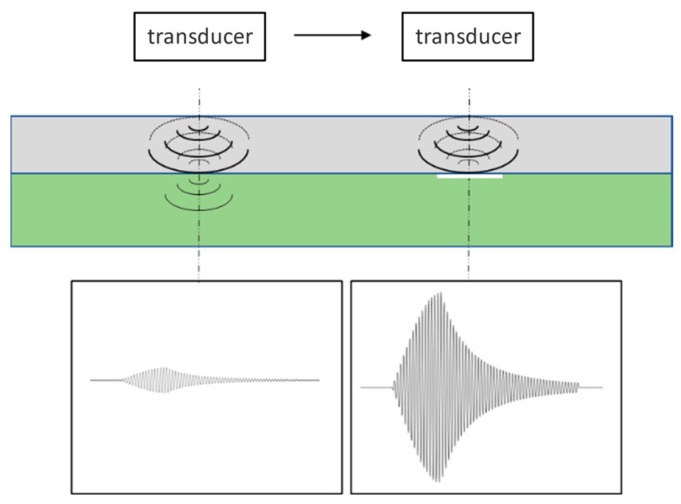
EMAR detection method for debonding defects.

When debonding occurs in adhesive structures, air defects hinder the propagation of acoustic energy between layers. Yalanskyy et al. [24] demonstrated that resonant wave modes are concentrated in the upper layer, while being weakened in the lower layer. As a result, the received signal predominantly consists of resonant wave modes from the upper layer, leading to a higher signal amplitude at this specific point. The EMAR detection method for identifying debonding defects is illustrated in Figure 2.

## 3. Finite Element Analysis

**Figure 3:**
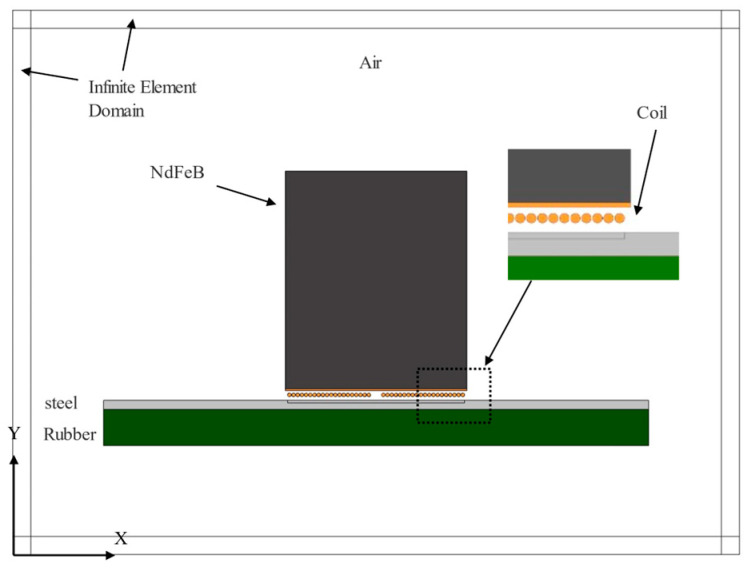
Model schematic of EMAR detection.

A two-dimensional modeling and analytical study of the EMAR detection method was conducted utilizing finite element analysis. The model includes an air domain, a permanent magnet, a coil, and the test piece, as illustrated in Figure 3. In this model, the magnet measured 10 mm × 12 mm, and the excitation coil consisted of a single layer of multiple turns of uniformly spaced circular wires. Each wire had a diameter of 0.2 mm, with a wire spacing of 0.03 mm, and there are 20 wires on both the left and right sides. The coil lift-off is 0.2 mm. The sample was composed of two rectangular layers representing the steel and rubber domains, with dimensions of 0.5 mm × 30 mm and 2 mm × 30 mm, respectively. The material parameters are shown in Table 1. These parameters were chosen according to real objects and default parameters provided by FEA software (Comsol v6.1). As for the boundaries, all boundaries in the simulation except the top surface are set as low-reflection boundaries, while the top is a free boundary. The low-reflection condition is used to significantly reduce artificial boundary reflections [25]. Meanwhile, the free boundary prevents reflected waves, minimizing interference from waves outside the detection path [26]. This combination of boundary conditions ensures accurate wave propagation and effective reflection control within the computational domain [27]. The air domain was mainly included to simulate the magnetic field distribution around the magnet, with the size of 40 mm × 30 mm. Simultaneously, a rectangular sleeve area was segmented from the four sides of the air domain and was set as an infinite element domain to simulate the far-field effect of the air.

The excitation signal was a sinusoidal pulse modulated by a Hanning window function, with a period of five cycles. A shorter period was chosen to minimize observation and reflection times, as longer periods did not contribute significantly to the experiment. The frequency was calculated using Equation (3) to determine the first-order resonant frequency of the steel layer, which is 3.2 MHz, and can be expressed as:

(4) $$an1(t)=\left\{\begin{array}{c} 10sin(2\pi ft)(1-\cos\left(\frac{2\pi ft}{5}\right)),\;0<x<5T\\ \;\;\;\;\;\;\;\;\;\;\;\;\;\;\;\;\;\;\;\;\;\;\;\;\;\;\;\;\;\;\;\;\;\;\;\;\;\;\;\;\;\;\;\;\;\;\;\;\;\;\;\;\;\;\;\;\;0\;,\;5T<x<100T \end{array}\right.$$

In the transient study module, the output time step of the model was set to one-tenth of the excitation signal period, with a total output duration of 20 μs. In the frequency domain study module, the frequency domain range was set to from 0.1 MHz to 10 MHz, with an interval of 0.1 MHz.

**Figure 4:**
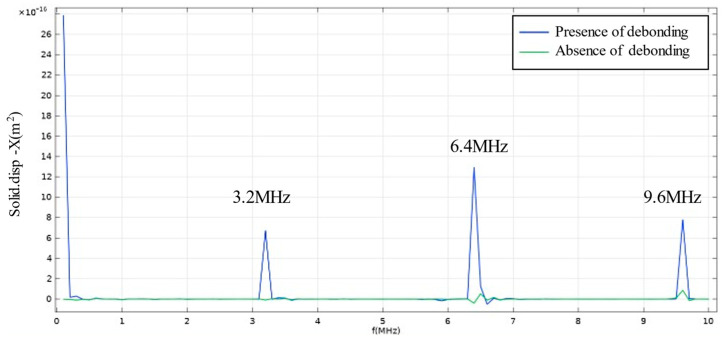
Frequency response of defective and non-defective models.

The frequency domain responses of both defective and defect-free models are illustrated in Figure 4, where the receiving probe was placed on the upper surface of the sample. It can be observed that the defective model exhibits a higher resonance peak compared to the non-defective model. The frequencies corresponding to these resonance peaks represent the first-order and higher-order resonance frequencies of the steel layer in the sample.

**Figure 5:**
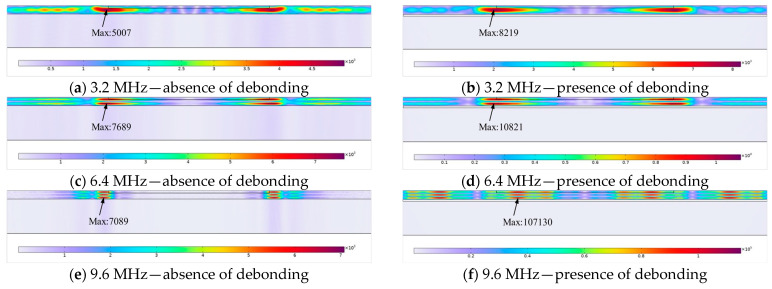

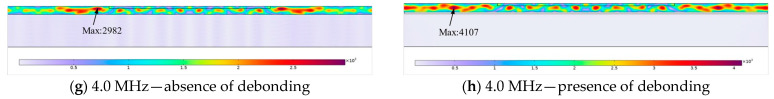
Resonance at different frequencies and different defect models.

The pressure contour maps at different frequencies for both defective and defect-free models are displayed in Figure 5, where the values in the figure are the peak pressure (N/m^2^). At an excitation frequency of 3.2 MHz, the wavelength of the shear wave in the steel layer is 1 mm. At this frequency, the excited shear or reflected shear waves at the air/steel interface constructively interfere with the reflected shear waves at the steel/rubber interface, resulting in waves with the same phase, as shown in Figure 5a,b. As the number of cycles increases, the first echo overlaps with additional reflected echoes, which in consequence leads to a higher amplitude. When the excitation wave frequency reaches the second-order or higher resonant frequencies, the wavelength of the shear wave in the steel layer decreases, inducing an increase in the corresponding wave number. At this point, the excited or the reflected shear waves at the air/steel interface continue to constructively interfere with the reflected shear waves at the steel/rubber interface, maintaining the same phase, as depicted in Figure 5c–f.

When the excitation frequency deviates from the resonant frequency, the phase difference between the excitation shear wave and the reflected wave alters. Instead of complete constructive interference, partial destructive interference occurs, resulting in an attenuation in wave amplitude. In Figure 5a,h, pressure contour maps are plotted for excitations at 3.2 MHz and 4 MHz, respectively, for the same sample. It is evident that significant resonance occurs within the steel layer at the excitation frequency of 3.2 MHz. Raising the excitation frequency to 4 MHz leads to a non-integer number of wavelengths within the steel layer, disrupting the resonance conditions and diminishing the amplitude. The closer the excitation frequency is to the resonant frequency, the more pronounced the resonance becomes; conversely, the further the excitation frequency is from the resonant frequency, the weaker the resonance effect. Comparing the resonance conditions at the same frequency across different defect models in Figure 5, it is observed that the presence of air defects impedes the propagation of acoustic energy at the interface. This decreases the acoustic energy in the lower structure while increasing the acoustic energy in the upper structure, thereby, the response of the defective model is more prominent than that of the non-defective model under resonance conditions.

**Figure 6:**
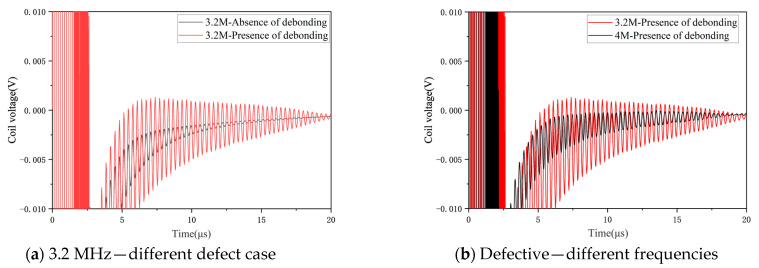
Coil voltage signals for different defect cases at different frequencies.

The time-domain transient study coil voltage is shown in Figure 6. At the same frequency, the return coil voltage amplitude of the defective model is significantly higher than that of the non-defective model. After filtering and removing the main shock, the peak-to-peak value of the non-defective model is 0.0076 volts, while the defective model exhibits a peak-to-peak value of 0.0148 volts, representing a 94% boost. Furthermore, in the defective model, the return coil voltage amplitude at the resonant excitation frequency is also notably higher than when the excitation frequency deviates from the resonance. At an excitation frequency of 4 MHz, the peak-to-peak value is 0.0057 volts, whereas at 3.2 MHz, it is 0.0148 volts, marking a 159% increase.

## 4. Experiment and Analysis

### 4.1. Experiment Systems and Samples

**Figure 7:**
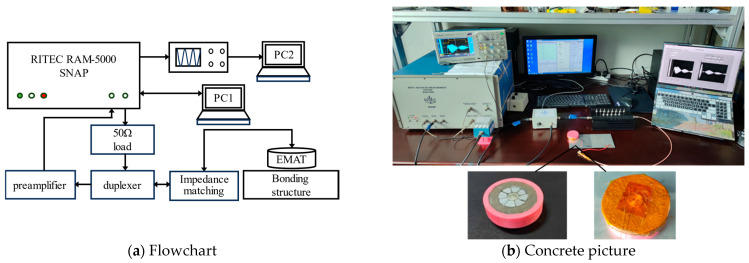
The EMAR experiment system.

The EMAR experiment system, shown in Figure 7, consisted of the RITEC RAM-5000 SNAP, EMAT, impedance matching network, duplexer, preamplifier, oscilloscope, and PC.

**Figure 8:**
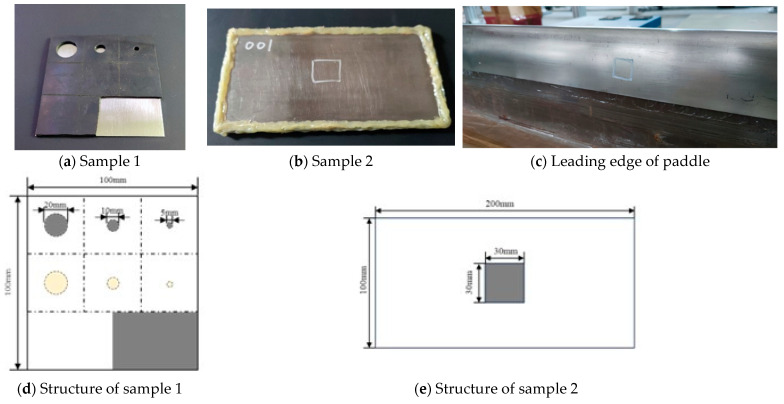
Pictures of samples.

The permanent magnets employed in the experiment were toroidal Halbach array magnets. It provided a higher bias field strength and conversion efficiency than traditional EMAT, by that enhanced excited ultrasonic signal [28]. Therefore, its higher bias field and magnetic flux density can enhance the amplitude of the excited signal without proportionally increasing noise, thereby improving the signal-to-noise ratio. Additionally, it allows for a more compact EMAT design without sacrificing strength, making it a viable option for field inspections in space-constrained environments. The coils were densely wound helical coils with a wire diameter of 0.2 mm, resulting in a total coil diameter of approximately 10 mm, matching the size of the center magnet in the toroidal Halbach array. To address the insulation and coil self-oscillation, the exposed area of the magnet in contact with the coil was coated with copper foil, and the coil was wrapped with high-temperature insulating tape, while the coil was then secured to the magnet’s center with adhesive tape. The clad-iron/composite structural layer of the leading edge of the helicopter paddle blade is a thin-walled adhesive structure, which consists of two flat-plate type simulation specimens and one leading edge of paddle, as shown in Figure 8.

The three test samples consist of a 0.5 mm thick steel layer and a 2 mm thick rubber layer. In sample 1, the bulge-type debonding was simulated by removing the rubber portion; meanwhile, the tight/inclusion type debonding was simulated by inserting a polytetrafluoroethylene (PTFE) film between the steel and rubber layers. The defects were shaped in three sizes, with diameters of 20 mm, 10 mm, and 5 mm. As for sample 2, debonding defects measuring approximately 30 mm × 30 mm were prefabricated by omitting the bonding agent. The paddle components contained natural defects formed during the manufacturing process.

The fabrication process for test samples involved a meticulous process to ensure the integrity of the bonding interface. For sample 1, initially, the surfaces were treated with acetone and 800-grit sandpaper. Then, the bonding interface was thoroughly wiped with industrial anhydrous ethanol and air-dried for 20 min. Figure 8d,e depicts the pre-set debonding defects, where white areas represent well-bonded areas, gray indicates removed rubber, and yellow marks the PTFE on one side. The adhesive was acrylic resin glue. It was then mixed and applied evenly, followed by a 24 h curing period at room temperature. In comparison, sample 2 was fabricated by the helicopter division of the aerospace industry; however, the exact steps are unknown.

**Figure 9:**
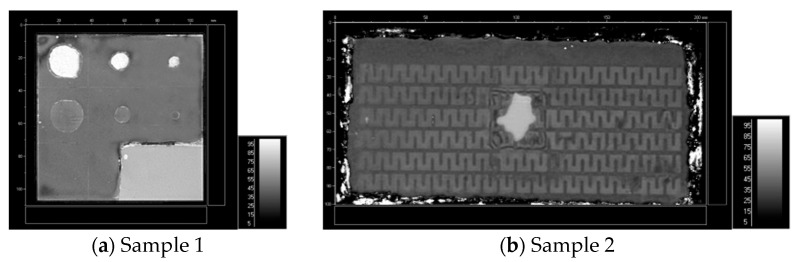
F-SCAN imaging results of samples 1 and 2.

In verifying the validity of the test samples fabrication, a water-immersed ultrasonic feature scanning imaging system (F-SCAN) was involved in the experiments to detect and image the samples, as shown in Figure 9. In Figure 9a, the image clearly distinguished the blister-type debonding in sample 1. Nevertheless, the tightly adhering debonding area appeared nearly identical to the well-bonded area, which can only be approximately distinguished from the debonding area contour. This was attributed to the minimal difference in reflection coefficients between the longitudinal wave’s direct incidence on the rigid joint interface and the slip interface. The water immersion ultrasonic detection of the two types of samples indicated that defective regions were markedly different from the rest of the image, presumably owing to the irregular shapes provoked by the pre-manufactured debonding defects.

### 4.2. Results of Debonding Detection Based on EMAR

**Figure 10:**
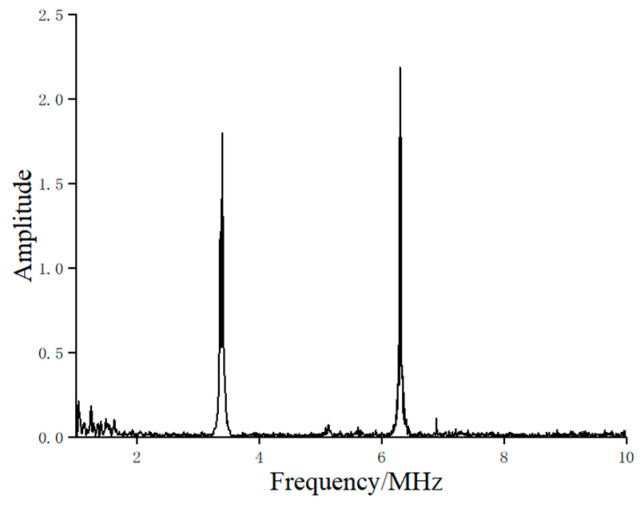
The resonance spectrum of sample swept by RITEC RAM-5000 SNAP.

The resonance spectrum of the sample was obtained using the sweep function of the RITEC (USA) RAM-5000 SNAP, as shown in Figure 10. It was observed that the resonant frequencies exhibited high amplitudes, while the non-resonant frequencies had significantly lower amplitudes, which can be distinctly differentiated. This included the first-order resonant frequency at 3.4 MHz.

**Figure 11:**
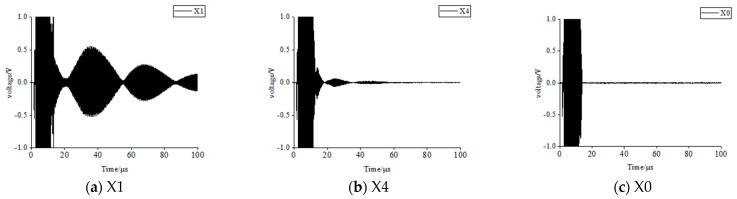
Time-domain signal in different bonding of sample 1.

In sample 1, the blister-type debonding defects were labeled X1 through X3 in descending order of size. The tight-contact debonding defects were also labeled X4 through X6 in the same order, and the well-bonded area was labeled X0. The time-domain signals are presented in Figure 11. The amplitude in the figure represents the intensity of the echo signal, which can also indicate the severity of the debonding (i.e., type). However, the exact size of the defect requires a complete scan to be determined. The resonant echo in the X1 area was distinct and exhibited a high amplitude. In the X4 area, PTFE was used to simulate close contact defects on the steel surface. The low surface tension and chemical inertness of PTFE created a relatively smooth, low-adhesion interface at the debonding site, which typically represented an air or PTFE surface. Compared to the strong debonding air interface in the X1 area, the debonding interface in the X4 area allows more transmission and less reflection of sound waves, resulting in smaller and lower amplitude resonant echoes.

Time-domain signals are widely used to evaluate the bonding state at interfaces; however, this measurement is prone to interference from electrical noise, particularly in low signal-to-noise ratio conditions. In addition, as opposed to the conventional pulse-echo signal, the resonance signal is continuous, with a long duration, and is characterized by its concentrated frequency-domain distribution. Therefore, two commonly used frequency domain signal processing methods are analyzed here for comparison.

**Figure 12:**
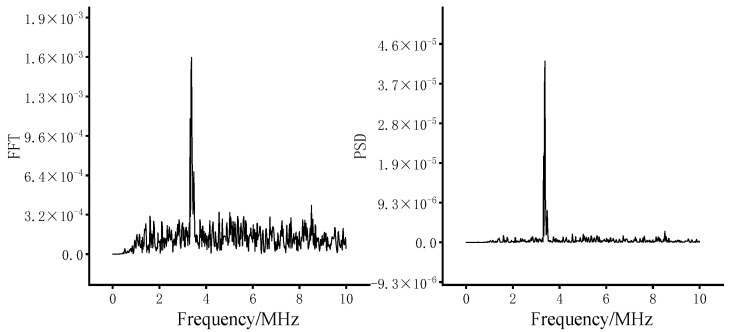
FFT and PSD of the resonant echo over a period on the time-domain signal of X6.

The FFT is widely employed in signal processing to convert a time-domain signal from the time-domain into the frequency domain, providing insight into the signal’s amplitude at various frequencies. The PSD is commonly used to analyze the spectral characteristics of a signal. It is considered the Fourier transform of the autocorrelation function, especially useful in random signal and noise analysis. PSD can also determine the primary frequency components and the power distribution of a signal. The FFT and PSD of a resonant echo over a period for the time-domain signal in X6 are shown in Figure 12. Due to the autocorrelation analysis performed in the PSD calculation, the non-dominant frequency signals (i.e., noise) are much lower in their amplitude spectrum compared to the FFT, resulting in a significantly higher signal-to-noise ratio. This is advantageous in identifying small defects.

**Figure 13:**
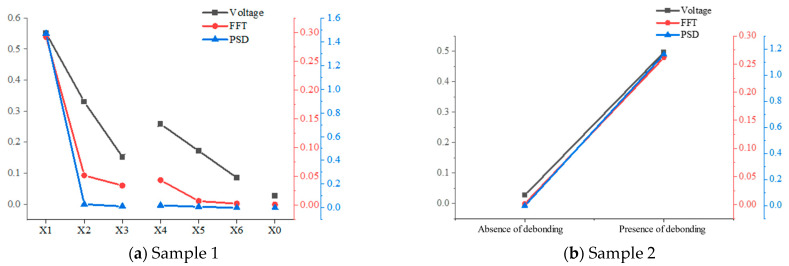
Signal amplitude for different bonding states of flat-plate objects.

The time-domain signal, FFT, and PSD amplitudes for different bonding states in the flat plate test sample are displayed in Figure 13, Table 2 and Table 3. All three methods effectively distinguish different bonding states based on their amplitudes. In the same sample, signal amplitude increases as bonding quality decreases or as the size of the debonding defect grows. When comparing the signal amplitudes across bonding states, the PSD amplitude exhibits the most pronounced change, followed by the FFT and the time-domain signal. This occurs because the resonance signal is the result of multiple superimposed echoes, and changes in the bonding state impact both the echo amplitude and the duration of the resonance echo, altering the shape of the resonance echo. These changes significantly influence the PSD and FFT amplitudes. The PSD amplitude is proportional to the square of the FFT result [29], which explains why it shows greater sensitivity to changes in the bonding state than the FFT and is more responsive to variations in bonding quality.

**Figure 14:**
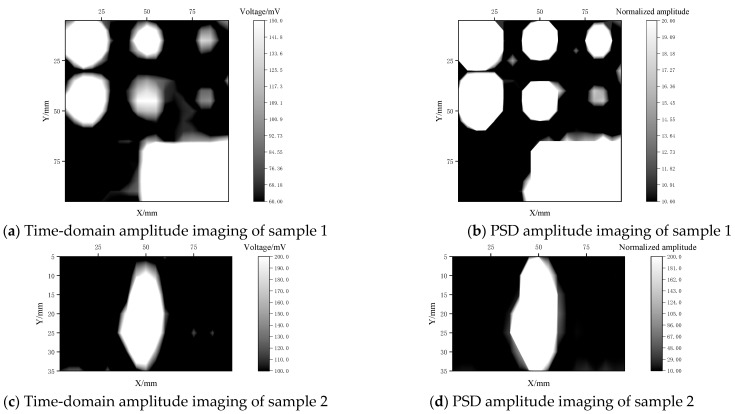
Amplitude imaging of plate samples 1 and 2.

In the experiment, sampling was conducted every 5 mm in both the X and Y directions, and the time-domain and PSD amplitudes at each point were recorded in real time. This resulted in 19 × 19 and 19 × 7 grid maps, as shown in Figure 14 after gray-scale imaging. To facilitate observation and comparison, the PSD amplitudes were normalized to their minimum value.

It is evident that EMAR detection technology enables both the time-domain amplitude and the PSD amplitude of the resonance echoes to effectively discriminate between debonding and well-bonded areas in the image. Compared to the direct incidence longitudinal wave detection results displayed in Figure 9, shear waves evince a stronger echo amplitude response to tight-contact defects in bonding areas than longitudinal waves. Imaging is less pronounced in debonding areas compared to well-bonded areas. Additionally, PSD amplitude imaging is more sensitive to changes in bonding state than time-domain amplitude imaging.

### 4.3. Quantitative Analysis of Debonding Defects Location

The sample comprised a steel/rubber double-layer material, and the primary defects were debonding defects at the steel/rubber interface, with the depth of the defects determined at the interface. In terms of horizontal positioning, EMAT was employed to detect the direct incidence of the test sample, and the horizontal location of the defects was confirmed by observing the defective echoes in the time-domain signal. Once the interface debonding location was identified based on the defect echo, the interface debonding defects were quantified using a length measurement method.

In Table 4, $∆x_{1}$ and $∆y_{1}$ represent the indicated lengths in the x and y directions, as measured by the 6 dB endpoint method for quantification, manually applied on the test sample via the EMAR method.$\;∆x_{2}$ and $∆y_{2}$ represent the indicated lengths of the debonding defects on the water immersion ultrasonic imaging map. Given the high resolution and high accuracy of the water immersion ultrasonic system, $∆x_{2}$ and $∆y_{2}$ are considered here as the actual defect dimensions, with $E_{x}=(∆x_{1}-∆x_{2})/∆x_{2}$ and $E_{y}=(∆y_{1}-∆y_{2})/∆y_{2}\;$ representing the errors.

The measured length quantitative results for debonding defects close to or larger than the probe coil size (10 mm diameter) does not differ significantly from the actual sizes, with a maximum error of about 10%. However, for debonding defects smaller than 5 mm, the measured lengths deviate significantly from actual sizes, with errors often exceeding 30%. This discrepancy is presumably attributed to the resolution limits of the large probe. When the transducer area of the probe exceeds the size of the defect, the ultrasonic beam becomes larger than the defect itself, increasing the effective interaction area between the ultrasonic waves and the defect. Research has validated this effect, demonstrating that the size of the probe influences detection and that using a transducer with a smaller aperture enhances defect resolution [30]. Therefore, this limitation hampers accurate localization of small defects boundaries, causing overestimation of their sizes.

The resolution and quantification of debonding defects by EMAR detection technology are constrained by the transducer’s area size, resulting in significant quantification errors for defects smaller than the transducer area. Further research and development of smaller electromagnetic ultrasonic transducers or focused probes is required to enhance the resolution of shape and size for small defects. However, this undoubtedly poses significant challenges at present. Another approach is to adopt more precise methods for defect quantification and localization. The 6 dB endpoint method is the optimal choice for defect waves, while the significance of resonance assignments is limited.

### 4.4. Experiments on Detection of Blade Leading Edge

**Figure 15:**
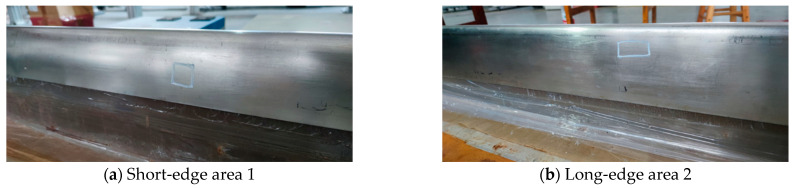
Leading edge of the paddle.

To further verify the effectiveness of EMAR detection on the leading edge of the paddle, signal sampling imaging was also performed on the structure in the experiment. The object is shown in Figure 15, which is a U-shaped structure, with its two end surfaces referred to as the short-edge end surface area 1 and the long-edge end surface area 2, according to the extension length.

**Figure 16:**
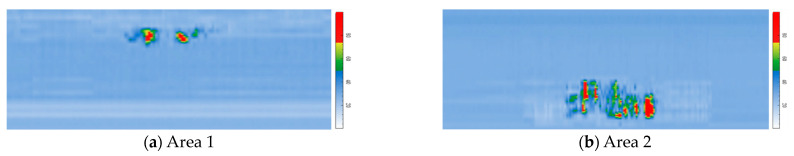
Phased-array ultrasonic scanning results in debonding at the leading edge of the blade.

Prior to signal sampling, phased-array ultrasonic technology was used to image the debonding defects present in the clad-iron structure as a reference. In the experiment, the CTS-PA32 phased-array ultrasonic detector and the corresponding 5M32-array flexible phased-array probe were employed to perform time-based scanning imaging of the clad-iron objects, and the results are shown in Figure 16. Phased-array ultrasonic technology clearly discriminates between defective and defect-free areas in the C-scan imaging through the time-domain amplitude. The debonding defects in the clad-iron objects were mostly irregular in shape, with a single debonding area often composed of numerous smaller debonding defects.

**Figure 17:**
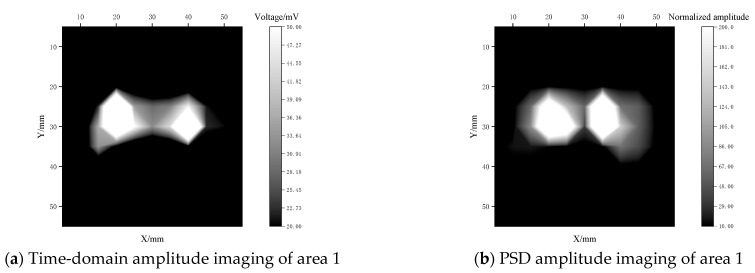

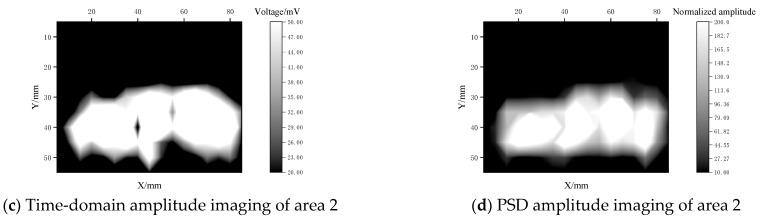
Amplitude imaging of leading edge of paddle.

Manual sampling of the leading edge of the paddle was conducted with a lift distance of 0.5 mm, and horizontal sampling spacing of 5 mm, to obtain 11 × 11 and 11 × 17 grid images, as shown in Figure 17. The EMAR detection technology productively distinguished between the well-bonded areas and the debonding defects in the clad-iron objects at the leading edge of the paddle. The variation in the shapes of time-domain magnitude imaging and PSD magnitude imaging is related to the echo superposition principle previously described. Both the time-domain signal amplitude and the frequency-domain PSD amplitude serve as parameters for assessing the bonding quality.

In Table 5, ∆*x*_1_ and ∆*y*_1_ represent the indicated lengths in the *x* and *y* directions, respectively, using the 6 dB manual endpoint quantification method via EMAR on the clad-iron blade’s leading edge. ∆*x*_2_ and ∆*y*_2_ represent the indicated lengths of debonding defects on the phased array imaging map. The defects errors are calculated as $E_{x}=(∆x_{1}-∆x_{2})/∆x_{2}$ and $E_{y}=(∆y_{1}-∆y_{2})/∆y_{2}$.

Considering the two debonding areas with shorter and longer sides as an example, it was concluded that the EMAR detection provides good quantitative results for the clad-iron leading edge of the rotor blade with a maximum error of about 15%.

In comparison with phased array detection, the experiments showed that EMAR technology produced more complete defect images, with lower identification loss and minimal error. Its inherently non-contact nature and strong adaptability to complex surfaces were also evident. Therefore, it can be inferred that the EMAR detection technique can effectively evaluate bonding quality in thin-walled metal/non-metal adhesive structures.

Based on the above discussions, we propose several potential optimizations. First, it will be important to address quantitative errors in subsequent studies to ensure they do not impact qualitative analyses. Additionally, optimizing the dimensions of the EMAT system can enhance performance by selecting higher-quality magnets. These steps will help improve the accuracy and reliability of our findings in future research.

## 5. Conclusions

To address the stringent demand for debonding defects detection in thin-walled adhesive structures, such as the clad-iron/rubber layers on the leading edge of helicopter rotor blades, the application of EMAR was proposed.

The generation mechanism of EMAR was investigated through theoretical analysis, and its advantages were further demonstrated via finite element analysis. EMAR detection was then applied to both test objects and real rotor blade components with thin-walled adhesive structures.

EMAR detection using shear wave excitation proved effective in identifying debonding defects, particularly tight-contact debonding defects that are challenging to detect with longitudinal wave inspection. The minimum detectable defect diameter was 5 mm.

EMAR detection can accurately locate and quantify debonding defects; the size limitation of the transducer’s active area results in higher quantitative error for defects smaller than the active area.

Finally, sampling and analysis were conducted on different clad-iron structures of the leading edge of the rotor blade. Compared with the phased-array ultrasonic scanning results, EMAR detection technology effectively identified the presence of debonding defects, with a maximum quantitative error of approximately 15%. It validates the effectiveness of EMAR detection technology for inspecting the clad-iron structure of the leading edge. Serval potential optimization is involved.

## Figures and Tables

**Table 1:** Material parameters.

Parameter	Value	Parameter	Value
Steel density	7930 kg/m^3^	Steel pressure wave velocity	5900 m/s
Steel shear wave velocity	3200 m/s	Rubber density	930 kg/m^3^
Rubber pressure wave velocity	1500 m/s	Rubber shear wave velocity	600 m/s
Copper relative permeability	1	Copper conductivity	5.998 × 10^7^ S/m
Copper relative dielectric constant	1	Relative permittivity of permanent magnet	1
Permanent magnet recoil permeability	1.05	Remanent magnetic flux density of the permanent magnet	1.21 T

**Table 2:** Signal amplitude for different bonding of sample 1.

	Time-Domain Signal	Normalized Result (Based on X_0_)	FFT	Normalized Result (Based on X_0_)	PSD	Normalized Result (Based on X_0_)
X1	0.55114	20.972	0.292	213.139	1.47	124,576.271
X2	0.33021	12.565	0.0518	37.810	0.0302	2559.322
X3	0.15223	5.793	0.03413	24.915	0.0124	1050.847
X4	0.25827	9.828	0.0437	31.898	0.0206	1745.763
X5	0.17208	6.548	0.00732	5.343	0.0089	754.237
X6	0.08493	3.232	0.00285	2.080	1.5 × 10^−4^	12.712
X0	0.02628	1.000	0.00137	1.000	1.18 × 10^−5^	1.000

**Table 3:** Signal amplitude for different bonding of sample 2.

	Time-Domain Amplitude	FFT	PSD
Absence of debonding	0.02717	0.0015	3.92 × 10^−5^
Presence of debonding	0.49655	0.262	1.16

**Table 4:** Quantitative data of samples 1 and 2.

	$∆x_{1}$/mm	$∆x_{2}$/mm	$E_{x}$	$∆y_{1}$/mm	$∆y_{2}$/mm	$E_{y}$
X1	18.4	20.2	−8.9%	18.0	20.4	−11.8%
X2	8.6	9.6	−10.4%	9.6	10.4	−7.7%
X3	7.2	5.6	28.6%	7.0	5.2	34.6%
X4	18.6	19.6	−5.1%	19.0	19.8	−4.0%
X5	9.4	10.6	−11.3%	10.0	9.8	2.0%
X6	7.4	5.4	37.0%	7.0	4.8	45.8%
Sample 2	20.2	21.6	−7.4%	25.0	26.2	−4.5%

**Table 5:** Quantitative data of areas 1 and 2.

	$∆x_{1}$/mm	$∆x_{2}$/mm	$E_{x}$	$∆y_{1}$/mm	$∆y_{2}$/mm	$E_{y}$
Area 1	17.8	20.2	−11.9%	9.6	10.8	−11.1%
Area 2	56.6	63.2	−10.4%	18.2	21.2	−14.2%

## Data Availability

The original contributions presented in the study are included in the article, further inquiries can be directed to the corresponding author.
